# The burden of hepatitis C virus infection in Punjab, India: A population-based serosurvey

**DOI:** 10.1371/journal.pone.0200461

**Published:** 2018-07-26

**Authors:** Ajit Sood, A. Suryaprasad, A. Trickey, S. Kanchi, V. Midha, M. A. Foster, E. Bennett, S. Kamili, F. Alvarez-Bognar, S. Shadaker, V. Surlikar, R. Garg, P. Mittal, S. Sharma, M. T. May, P. Vickerman, F. Averhoff

**Affiliations:** 1 Dayanand Medical College, Ludhiana, Punjab, India; 2 Division of Viral Hepatitis, Centers for Disease Control and Prevention, Atlanta, GA, United States of America; 3 Population Health Sciences, University of Bristol, Bristol, United Kingdom; 4 MSD India Pvt. Ltd., Mumbai, India; 5 Merck & Co. Inc., Kenilworth, NJ, United States of America; 6 CDC Foundation, Atlanta, GA, United States of America; 7 Guru Gobind Singh Medical College and Hospital, Faridkot, Punjab, India; 8 Mittal Liver and Gastroenterology Centre, Patiala, Punjab, India; 9 College of Nursing, All India Institute of Medical Sciences, Rishikesh, Uttarakhand, India; Defence Research Laboratory, INDIA

## Abstract

**Introduction:**

Hepatitis C virus (HCV) infection prevalence is believed to be elevated in Punjab, India; however, state-wide prevalence data are not available. An understanding of HCV prevalence, risk factors and genotype distribution can be used to plan control measures in Punjab.

**Methods:**

A cross-sectional, state-wide, population-based serosurvey using a multi-stage stratified cluster sampling design was conducted October 2013 to April 2014. Children aged ≥5 years and adults were eligible to participate. Demographic and risk behavior data were collected, and serologic specimens were obtained and tested for anti-HCV antibody, HCV Ribonucleic acid (RNA) on anti-HCV positive samples, and HCV genotype. Prevalence estimates and adjusted odds ratios for risk factors were calculated from weighted data and stratified by urban/rural residence.

**Results:**

5,543 individuals participated in the study with an overall weighted anti-HCV prevalence of 3.6% (95% Confidence Interval [CI]: 3.0%–4.2%) and chronic infection (HCV Ribonucleic acid test positive) of 2.6% (95% CI: 2.0%–3.1%). Anti-HCV was associated with being male (adjusted odds ratio 1.52; 95% CI: 1.08–2.14), living in a rural area (adjusted odds ratio 2.53; 95% CI: 1.62–3.95) and was most strongly associated with those aged 40–49 (adjusted odds ratio 40–49 vs. 19–29-year-olds 3.41; 95% CI: 1.90–6.11). Anti-HCV prevalence increased with each blood transfusion received (adjusted odds ratio 1.36; 95% CI: 1.10–1.68) and decreased with increasing education, (adjusted odds ratio 0.37 for graduate-level vs. primary school/no education; 95% CI: 0.16–0.82). Genotype 3 (58%) was most common among infected individuals.

**Discussion:**

The study findings, including the overall prevalence of chronic HCV infection, associated risk factors and demographic characteristics, and genotype distribution can guide prevention and control efforts, including treatment provision. In addition to high-risk populations, efforts targeting rural areas and adults aged ≥40 would be the most effective for identifying infected individuals.

## Introduction

There are an estimated 70 million people living with hepatitis C Virus (HCV) infection around the world.[1] Persons living with HCV infection are at risk of developing liver cirrhosis and progressing to end stage liver disease and liver cancer (hepatocellular carcinoma).[2–7] Globally, an estimated 700,000 people die annually due to complications related to HCV infection.[8]

The World Health Organization (WHO) has set ambitious targets to eliminate HCV infection as a public health problem by 2030.[9] In order to achieve these targets, which include reduction of new infections by 90% and deaths by 65%, there is a need to increase prevention strategies and access to treatment. Treatment for HCV has improved dramatically with the addition of direct acting antivirals (DAAs), which are easy to take oral regimens that are highly effective, have minimal side effects, and achieve cure rates of over 90%.[10, 11] In order to establish effective prevention and treatment programs, there is a need to understand the epidemiology and burden of disease in the country or community. However, such data are lacking in many countries, particularly in lower and middle income countries which shoulder most of the burden.[12] There are significant geographical variations in prevalence patterns and genotype distribution globally,[12, 13] with populations in North America and Western Europe having anti-HCV prevalence rates generally less than 1%, while in some areas of Asia and the Middle East, prevalence rates exceed 5%.[1, 12–15] In India, where genotype 3 is thought to be most common,[16] population based studies on HCV infection prevalence are lacking, and the epidemiology is not well described. Some studies from India suggest the HCV prevalence may be low, however, there are significant variations within regions and sub-populations, with some studies demonstrating very high prevalence rates.[15, 17]

Population HCV seroprevalence data are lacking in Punjab, a state in Northern India with an estimated population of 28 million.[18] A survey conducted in one district of Punjab in 2003 found a 5% anti-HCV positive rate; in this study, infection was associated with reuse of needles and syringes, history of surgery, and history of dental extraction.[19] Elevated rates of HCV infection have also been identified among high risk populations (eg. people who inject drugs [PWID]) in Punjab,[20, 21] which may reflect the growing epidemic of injection drug use, a high-risk behavior for HCV infection.[22]

Epidemiological assessment of the burden of disease and related risk factors in the state are essential for public health planning strategies to combat this disease. This study assessed the prevalence of HCV infection in Punjab and identify risk factors associated with the disease.

## Materials and methods

### Sample design

A cross-sectional seroprevalence survey was conducted in the state of Punjab, India during October 2013 –April 2014. Punjab is divided into three major geographical areas, Doaba, Majha, and Malwa which contain a total of 22 districts. The survey sample size was calculated to enable estimation of HCV prevalence among individuals age 5 years and older, using the statistical software PASS (NCSS, LLC. 2011 Kaysville, Utah, USA). For expected HCV prevalence of 5% with a 95% confidence interval (CI) of 4–6%, the effective sample size was estimated to include 1,924 households; and assuming a design effect of 2 and overall response rate of 70%, the target sample size was 5,500 individuals.

The study included testing for past and current HCV infection, past infections with hepatitis A virus and hepatitis E virus, and past or current infection with hepatitis B virus (HBV). Results will only be presented for HCV infection in this report. The sample size of 5,500 individuals was expected to be large enough to produce combined estimates with relative standard errors of 10–20%. For stratified analysis, a minimum sample size of 1,000 per strata was expected to produce estimates with relative standard errors of 25% or less. Estimates based on relative standard errors >25% are considered unreliable.

The survey used a multi-stage stratified cluster sampling design using 2011 Punjab Census data,[18] and 10 of the 22 districts in Punjab were selected with probability proportionate to size. In rural areas, 22 sub districts and 87 villages were selected proportionate to size, and 813 households were systematically selected in groups of five. To ensure the selection of a sufficient number of households in rural areas, villages with fewer than five households were excluded, and villages with 5–49 households were combined with neighboring villages, for a minimum of 50 households per sampling unit. In urban areas, 13 sub districts and 41 wards were selected proportionate to size; 1 census enumeration block of 150–200 households was randomly selected per ward; and 586 households were systematically selected in groups of five. For large sampling units, villages and census enumeration blocks with 500 or more households were divided into three or more segments and two segments were selected proportionate to size.

All household residents and guests 5 years of age and older of selected household residents who stayed at the household the previous night were eligible to participate in the study. Selected adults ≥18 years of age who provided informed consent, and children age 5–17 years who provided assent and informed parental/guardian consent were included. Pregnant women were included, since participation in the study did not pose any risk to the mother or her unborn child. Individuals under 5 years of age and those who did not provide consent or assent were not included. No replacement was made if selected household was not available during data collection.

### Data collection

Trained survey teams consisting of a doctor, a phlebotomist, a nurse and a social worker visited selected households and administered the survey questionnaire, after obtaining informed consent and assent from children willing to participate. The study questionnaire was administered as a face-to-face interview and inquired about socio-demographic data, medical history, lifestyle information, obstetric history (if applicable), and potential exposures to HCV, including healthcare and lifestyle associated exposures. Each completed questionnaire was reviewed in the field by the team doctor, and if inconsistencies or gaps were identified, an attempt to correct or fill in the missing information was made by revisiting the surveyed individual before leaving the cluster. Each completed interview was labeled with a bar code with a unique identifier.

After completing the interview, a blood sample of approximately 16ml was drawn in a serum separator tube and labeled with a barcode matching the interview form completed by the study subject. Within one hour of collection, the sample was centrifuged for 15 minutes at 3,000 revolutions per minute. Separated serum was pipetted into 2ml cryovials, which were also labeled with bar codes matching the study subjects. Up to eight aliquots of sample per subject were prepared and frozen at -80C. Specimens were shipped every 2 weeks to a central laboratory (Oncquest Laboratories Ltd) in Delhi for testing. All samples were tested for anti-HCV (Vitros Immunodiagnostic Anti-HCV, Johnson and Johnson Co., New Brunswick, NJ, USA) and all anti-HCV positive samples were tested for HCV RNA (COBAS® TaqMan® HCV Test, Roche, Indianapolis, IN, USA). All HCV RNA positive samples were genotyped by Linear Array HCV genotyping test (Roche, Indianapolis, IN, USA). Survey participants who tested positive for anti-HCV were considered infected with HCV, regardless of HCV RNA results. Survey participants who tested positive for anti-HCV and HCV RNA were considered to have current infection, and those that tested anti-HCV positive and HCV RNA negative were considered to have past infection. Specimens were also tested for hepatitis A virus, hepatitis B virus and hepatitis E virus markers of infection (methods and results not described in this report). Unused blood was disposed of as per healthcare waste management guidelines and all specimens were destroyed following completion of the study.

### Counseling and notification of test results

For consenting participants, pretest counseling and educational brochures on HCV transmission and prevention were administered prior to interview and venipuncture. Study participants were notified of their test results for HCV, HBV, HAV, and HEV infection or immunity by telephone and mail within three weeks of the interview date. Patients found to have current (active) HBV or HCV infection were offered post-test counseling by appointment. All participants were counseled about measures to prevent the risk of transmission of the various forms of viral hepatitis.

### Ethical considerations

The protocol for this study underwent approval from the Institutional Review Board (IRB) at Dayanand Medical College, Ludhiana, and the Merck Investigator Initiated Study Protocol-Review Committee (MISP-RC). Participation was voluntary and confidentiality was strictly adhered to during the survey. Written consent was documented by the study subject’s dated signature or thumbprint on a consent form along with the dated signature of the person who conducted the consent discussion. A copy of the consent form was given to the subject prior to participating in the survey. Consent forms were available in English, Punjabi and Hindi. If the subject was illiterate, a witness was present during the entire informed consent reading and discussion. Afterward, the subjects signed and dated the consent if literate, or a thumb impression was taken. The witness also signed and dated the consent form along with the study staff who read and discussed the consent. Children ≥5 years and <18 years of age provided assent in addition to having parental permission.

### Statistical methods

Analyses of the survey data were weighted according to the population sizes of the wards and villages estimated from the 2011 population census. This weighting was stratified by urban/rural status. The HCV prevalence was estimated for the state as a whole, by urban/rural residence, and by district. A χ^2^ test was used to examine whether the proportion of HCV RNA positive patients with each genotype differed by district. Participant characteristics and prevalence of HCV risk factors were tabulated for those testing positive for anti-HCV and those testing positive for HCV RNA. The variables included in these tabulations were district, age-group (5–18, 19–29, 30–45, 46–60, >60), sex, urban/rural status, household income in rupees (<20,000, ≥20,000), education status (never educated/primary education, middle/secondary, graduate/above), the number of injections received in the last 6 months (0, 1–3, 4–8, >8), who administered the last injection received (Medical Doctor, Registered Nurse/Medical Practitioner, Other/Unknown (including chemists and unlicensed practitioners), the number of lifetime blood donations (0, 1–3, 4–6, >7), the number of blood transfusions received (0, 1–3, >3), if ever received a permanent tattoo, if ever used injectable drugs, or had ever received renal dialysis. Proportions and numbers presented in the tabulations were weighted to represent the population surveyed. Tabulations were stratified by urban/rural status. We estimated the association of patient characteristics and HCV risk factors with HCV status using weighted logistic regression models for the total survey population, stratified by urban/rural status, and clustered by household. Age, the number of injections received in the last 3 months, the number of times the person had donated blood, the number of blood transfusions received were included in models as continuous variables. Results are presented as weighted unadjusted and mutually adjusted odds ratios (OR) of having a positive anti-HCV test, with 95% confidence intervals (CI). We also estimated the association of the year of the first blood transfusion received (grouped as before 2002, 2002 or later year, year unknown, and no blood transfusions received; of note, blood bank testing for HBV and HCV became mandatory in Punjab by law in 2002) with HCV status. We used the same mutually adjusted model as above, but instead of including the number of blood transfusions we included the year of first receiving a blood transfusion. A sensitivity analysis excluded participants under 18 years of age because some risk factors only applied to adults, and another sensitivity analysis (not stratified by urban/rural status) only included participants aged 40–59 years of age as these were the two highest prevalence age groups.

We examined the number of injections (categorized: 0, 1–3, 4–8, >8) received in the last 6 months by anti-HCV prevalence. We examined the relationship of cumulative number of different types of potential exposures found to be associated with anti-HCV prevalence by univariate analysis (including having a permanent tattoo, ever received a blood transfusion and received a medical injection within the last 6 months) and testing positive for anti-HCV. We used logistic regression to estimate the adjusted OR of anti-HCV positivity for number of risk factors (1, 2–3) compared with no risk factors.

## Results

There were a total of 5,548 individuals who agreed to participate in the study and completed the questionnaire, however, 5 lacked HCV testing results and were excluded, resulting in 5,543 subjects for inclusion in the analyses. The median age of our sample was 35 years (interquartile range 21, 50) while the largest age group was participants age 5–18 years (Table 1). Among the participants, there were more women (53.8%) than men (46.2%), and 62.4% resided in rural areas (Table 1). The majority of participants, 81.9%, lived in households with an income of less than 20,000 Indian rupees (about 300 US dollars) which is below the national average of 27,857 Indian rupees.[23] and 12.5% attended graduate school (Table 1).

**Table 1:** Weighted and unweighted participant demographic characteristics and prevalence of potential exposures and risk factors associated with Hepatitis C (HCV) infection, with percent testing positive for HCV antibodies (anti-HCV) and HCV-RNA cells.

Variables	Unweighted Population	Weighted Population	% with positive anti-HCV (95% confidence intervals)	% with HCV RNA (95% confidence intervals)
Overall	5543	100%	3.6% (3.0%, 4.2%)	2.6% (2.0%, 3.1%)
Age Group (years)				
5–18	1107	20.2%	0.7% (0.1%, 1.2%)	0.4% (0.0%, 0.8%)
19–29	1024	18.3%	1.7% (0.8%, 2.5%)	1.2% (0.5%, 1.9%)
30–39	998	18.0%	4.3% (2.9%, 5.7%)	3.1% (1.8%, 4.3%)
40–49	870	15.7%	6.2% (4.4%, 8.0%)	4.7% (3.1%, 6.2%)
50–59	721	13.0%	5.8% (3.9%, 7.7%)	4.5% (2.7%, 6.2%)
≥60	823	14.9%	4.3% (2.7%, 5.8%)	2.7% (1.4%, 3.9%)
Sex				
Female	3005	53.8%	3.2% (2.5%, 3.9%)	2.3% (1.7%, 2.9%)
Male	2538	46.2%	4.0% (3.1%, 5.0%)	2.8% (2.1%, 3.6%)
Region				
Urban	2083	37.6%	1.6% (1.1%, 2.2%)	1.0% (0.6%, 1.4%)
Rural	3460	62.4%	4.7% (3.8%, 5.7%)	3.5% (2.7%, 4.3%)
Household income (rupees)				
<20,000	4546	81.9%	3.8% (3.1%, 4.5%)	2.7% (2.1%, 3.3%)
≥20,000	997	18.1%	2.5% (1.2%, 3.7%)	1.9% (0.8%, 2.9%)
Education				
Never/Primary School	2114	37.7%	4.7% (3.6%, 5.8%)	3.8% (2.8%, 4.8%)
Middle/Secondary School	2735	49.8%	3.4% (2.6%, 4.1%)	2.1% (1.5%, 2.7%)
Graduate/Above	694	12.5%	1.1% (0.3%, 1.8%)	0.6% (0.1%, 1.2%)
No. injections in last 6 months				
0	3639	65.2%	3.1% (2.4%, 3.8%)	2.2% (1.7%, 2.8%)
1–3	1155	21.1%	3.8% (2.6%, 5.0%)	2.5% (1.6%, 3.4%)
4–8	461	8.3%	4.7% (2.5%, 6.9%)	3.7% (1.6%, 5.7%)
>8	288	5.4%	7.0% (3.5%, 10.4%)	5.0% (2.1%, 7.9%)
Last injection given by				
Medical Doctor	1149	20.4%	2.1% (1.2%, 2.9%)	1.3% (0.6%, 2.0%)
Registered Nurse/Medical Practitioner	3090	56.9%	4.4% (3.6%, 5.2%)	3.3% (2.5%, 4.0%)
Other/Unknown	1304	22.7%	2.9% (2.9%, 4.0%)	1.9% (1.0%, 2.9%)
Number of times blood donated				
0	4808	86.5%	3.6% (2.9%, 4.2%)	2.5% (1.9%, 3.0%)
1–3	528	9.8%	3.3% (1.5%, 5.1%)	3.2% (1.4%, 5.0%)
4–6	115	2.1%	4.6% (0.5%, 8.6%)	3.6% (0.0%, 7.3%)
≥7	92	1.7%	5.3% (0.2%, 10.3%)	2.4% (0.0%, 5.8%)
Number of transfusions received				
0	5175	93.6%	3.4% (2.8%, 4.0%)	2.4% (1.9%, 2.9%)
1–3	353	6.3%	5.9% (3.2%, 8.6%)	4.6% (2.2% (6.9%)
>3	15	0.2%	25.8% (0.0%, 53.7%)	25.8% (0.0%, 53.7%)
Received a permanent tattoo				
Yes	479	8.6%	5.2% (2.8%, 4.1%)	3.7% (1.9%, 5.4%)
No	5064	91.4%	3.4% (3.1%, 7.3%)	2.5% (1.9%, 3.0%)
Use of Injectable Drugs				
Yes	5	0.1%	25.1% (0.0%, 66.8%)	25.1% (0.0%, 66.8%)
No	5538	99.9%	3.6% (2.9%, 4.2%)	2.5% (2.0%, 3.1%)
Any dialysis				
Yes	26	0.4%	0.0% (0.0%, 0.0%)	NA
No	5517	99.5%	3.6% (3.0%, 4.2%)	2.6% (2.0%, 3.1%)

When we examined potential exposures for HCV infection, 34.8% of participants had received one or more medical injections in the previous 6 months in the weighted analysis. When asked who administered their last medical injection, 20.4% identified a medical doctor and 56.9% identified a registered nurse or registered medical practitioner (eg. medical care provider not having the qualifications/training of a medical doctor). For those who had received an injection in the last 6 months, 24% received it from a medical doctor, 71% from a registered nurse or registered medical practitioner and 5% from other sources (eg. chemist or pharmacist, unlicensed practitioner, or did not specify). Of the participants, 6.5% stated they had received at least one blood transfusion. Additionally, 8.6% of patients had received a permanent tattoo, while few (0.1%) participants admitted to using injectable drugs.

Overall, of the 5,543 persons tested for hepatitis C, 3.6% (95% CI: 3.0%, 4.2%) tested positive for anti-HCV (ever infected), and 2.6% (95% CI: 2.0%, 3.1%) tested positive for HCV RNA, indicative of current infection. Among the 138 that tested positive for RNA, 130 were successfully tested for genotype, the majority were classified as genotype 3 (61.2%), followed by genotype 1 (27.5%) and genotype 4 (11.3%). No participants in our study were found to have genotype 2. The proportions of RNA positive patients with each genotype differed by province (p = 0.038).

**Fig 1:**
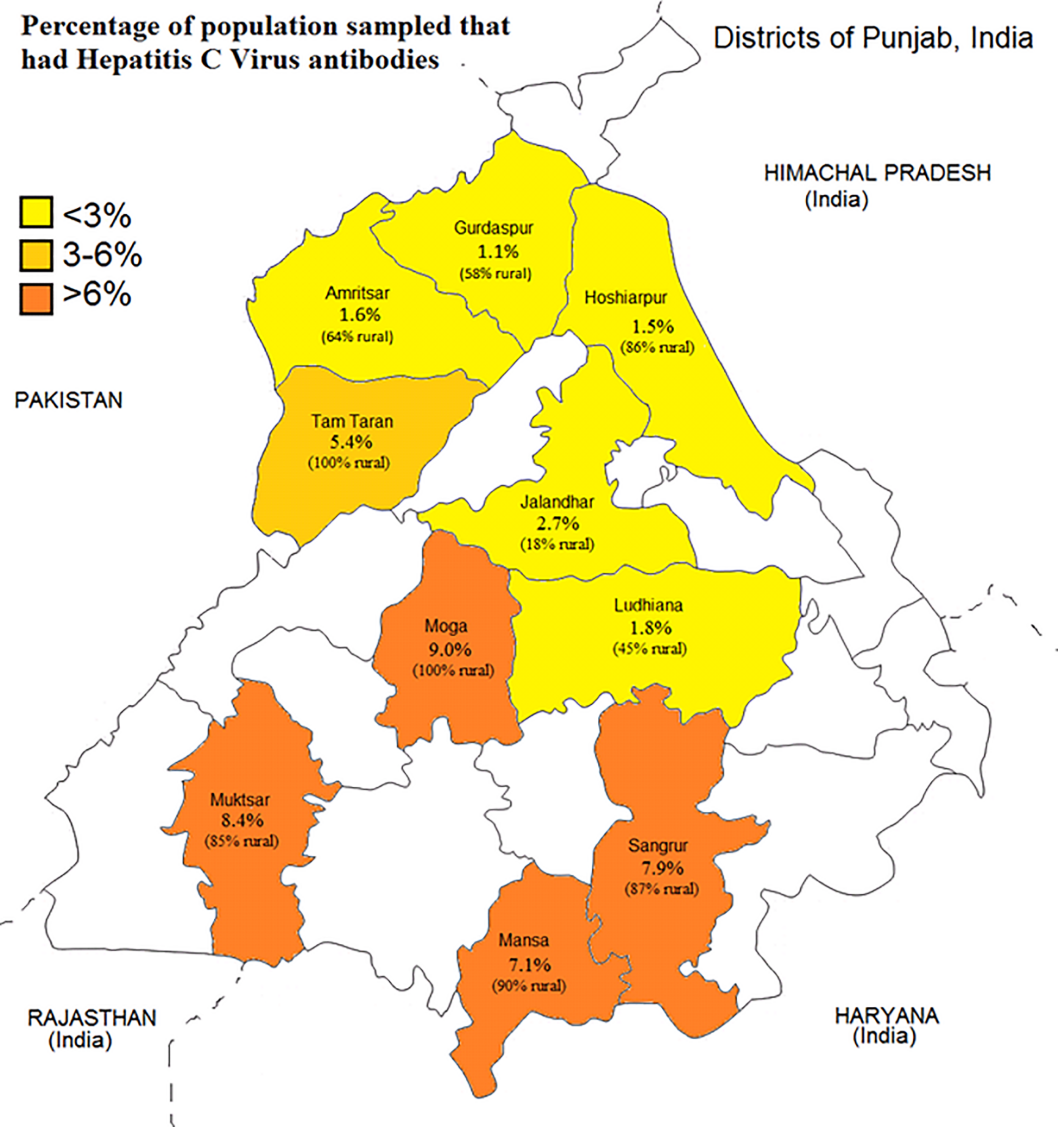
The percentage of participants sampled in each district that had Hepatitis C virus antibodies.

Anti-HCV prevalence was higher among rural residents (4.7% [3.8%, 5.7%]) than urban residents (1.6% [1.1%, 2.2%]) (Table 1). The proportion of persons testing positive for HCV differed by district, ranging from 1.1% in Gurdaspur to 9.0% in Moga (Fig 1); however, this study was designed to estimate prevalence for Punjab as a whole, not to estimate district level prevalence.

**Fig 2:**
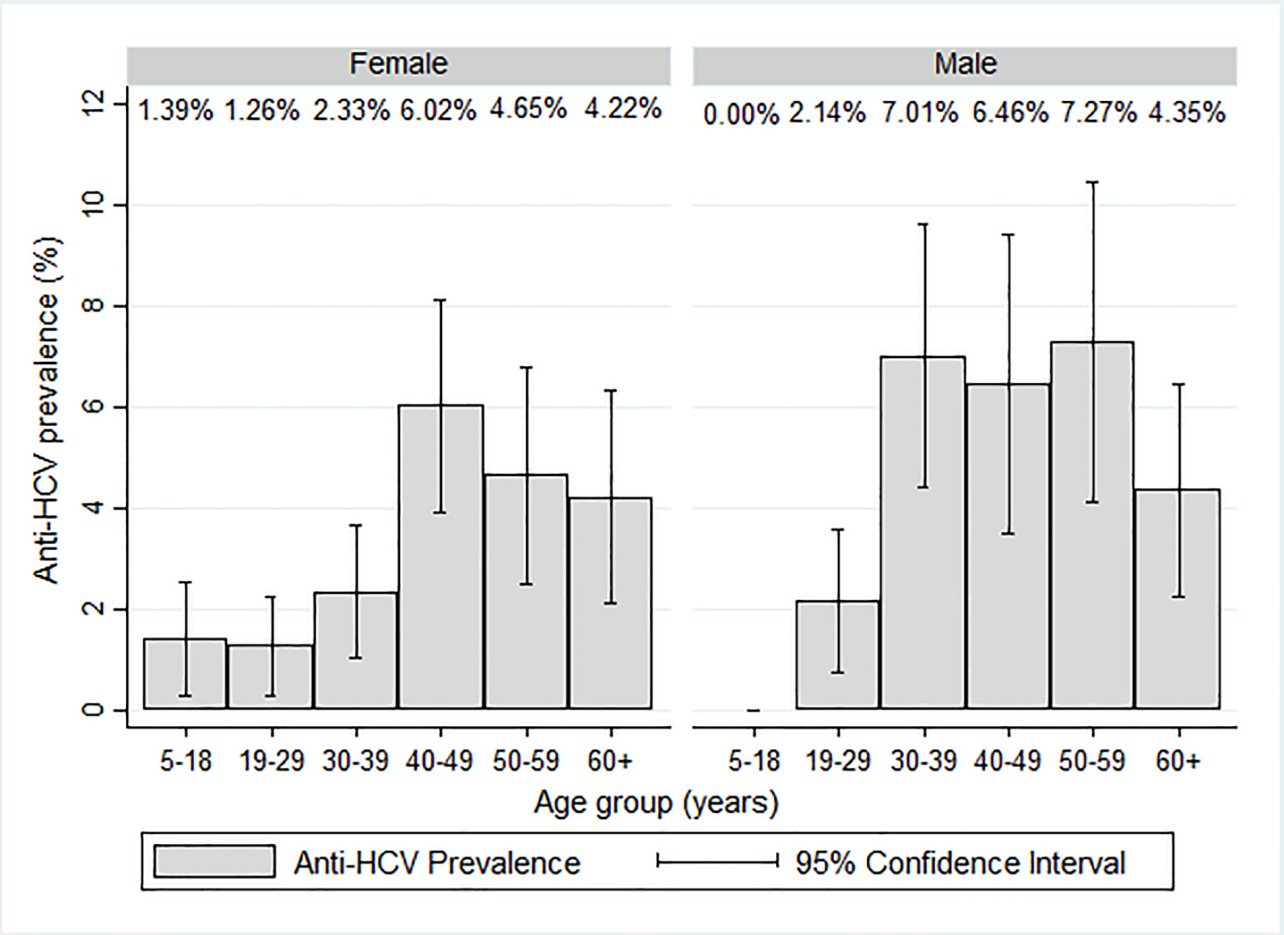
Prevalence of Hepatitis C antibodies (95% confidence interval) by age category and sex.

When we examined anti-HCV prevalence by age, we found prevalence increased with age up to 40–49 years where it peaked and then decreased with increasing age (Table 1). Overall, prevalence among men and women was similar, (Table 1), and when stratified by age, there were some small differences in seroprevalence by age groups among men and women (Fig 2). Anti-HCV prevalence also decreased with increasing educational attainment, and was lower among persons with higher income (Table 1).

**Fig 3:**
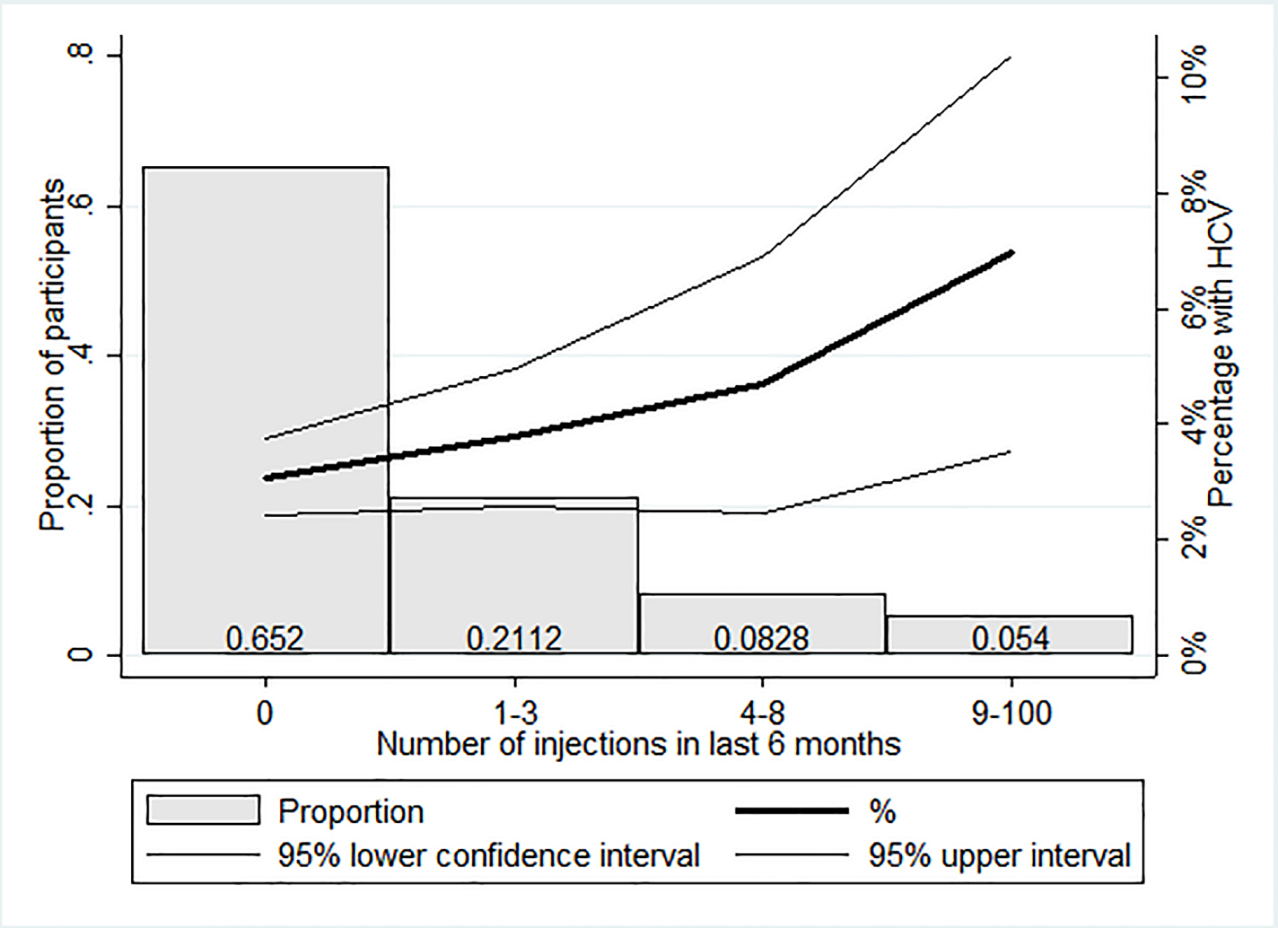
Prevalence of Hepatitis C antibodies by number of medical injections received in the last 6 months.

**Fig 4:**
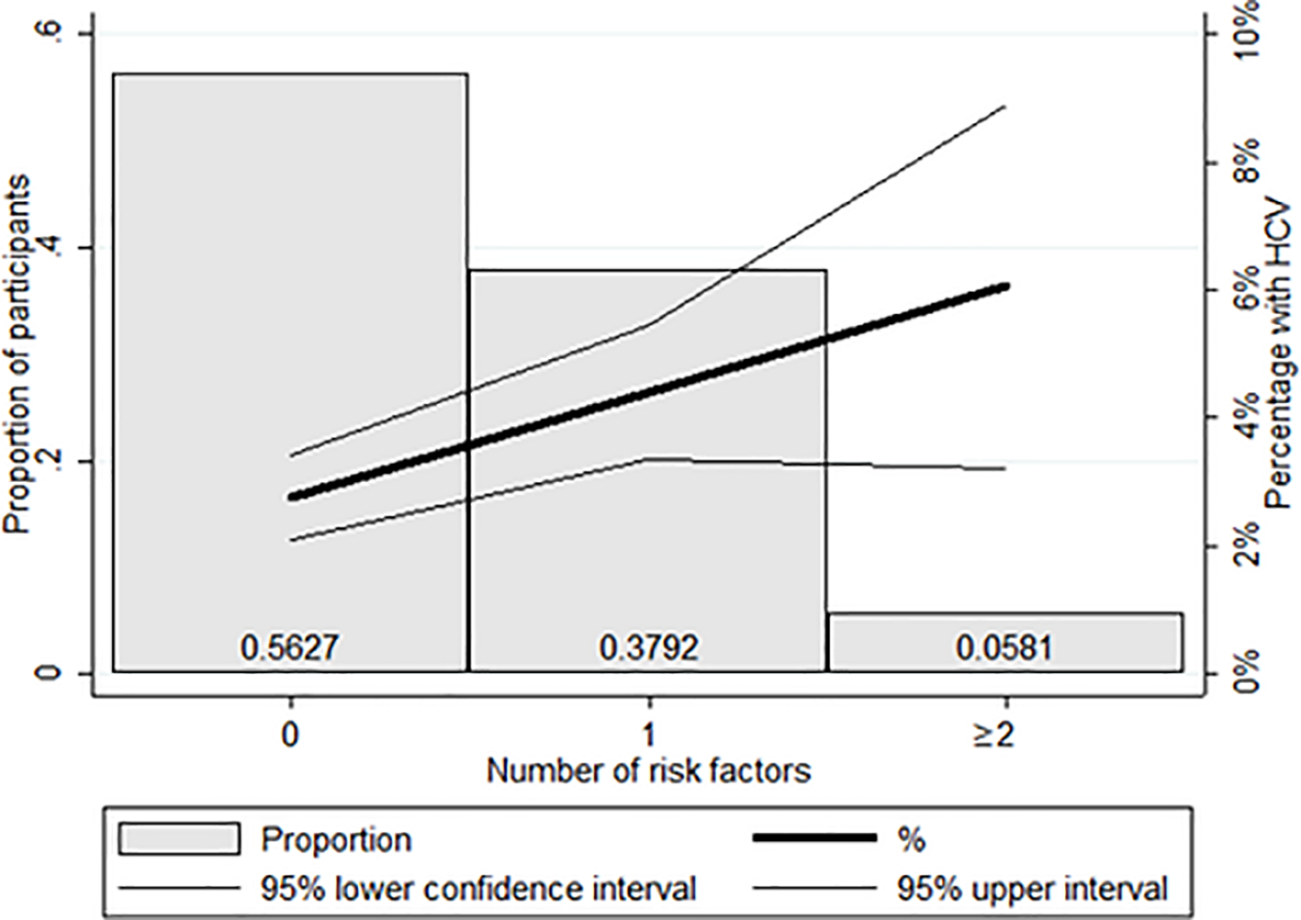
Prevalence of Hepatitis C antibodies by unique potential exposures (whether or not they had a permanent tattoo, whether they had ever received a blood transfusion and whether in the last 6 months they had received a medical injection).

When we examined the prevalence of anti-HCV antibody positivity by potential exposures and risk factors, we found that rates were higher as the number of injections received increased (see Fig 3), were highest for those whose last injection was administered by a nurse, registered medical practitioner, or other non–medical doctor, increased with the number of transfusions received, and also were higher among persons who had received a tattoo (Table 1). There were no HCV infections among persons who had a history of receiving dialysis (Table 1); however, the number persons associated with some of the exposures and risk factors, including dialysis (n = 26) and injection drug use (n = 5), was small. When we examined anti-HCV prevalence by the number of unique potential exposures, compared to persons without these potential exposures, the HCV prevalence increased as the cumulative number of unique exposures increased (Fig 4); the same analysis revealed that overall 43.7% of participants had one or more type of potential exposure.

When we examined demographic and potential exposures and risk factors in a multivariable model, calculating adjusted odds ratios, we found similar findings to our bivariate analysis: that testing positive for anti-HCV was associated with increasing age to age 40–49 years with the odds decreasing slightly for older age groups, being male, rural residence, lower educational attainment, and receipt of blood transfusions (Table 2). Results were similar when the analysis was restricted to adults (Table 3), and when the analysis was restricted to the two highest prevalence age groups– 40–49 and 50–59 years (Table 4). When we stratified the analysis by urban and rural residence, all of the associations, except educational attainment, persisted among rural residents, while among urban residents, only age remained a significant predictor. We did not find a difference in the likelihood of having HCV infection among those who received a blood transfusion before 2002 and those who received one during or after 2002.

**Table 2:** Weighted unadjusted and mutually adjusted odds ratios (OR) for having Hepatitis C antibodies (anti-HCV), by participant characteristics and risk factors, overall and stratified by urban/rural status.

	Total	Odds Ratio (95% Confidence Interval)		Urban	Odds Ratio (95% Confidence Interval)		Rural	Odds Ratio (95% Confidence Interval)	
Variables	Anti-HCV % (95% CI)	Unadjusted OR	Adjusted OR	Anti-HCV % (95% CI)	Unadjusted OR	Adjusted OR	Anti-HCV % (95% CI)	Unadjusted OR	Adjusted OR
Total	3.6% (3.0%, 4.2%)			1.6% (1.1%, 2.2%)			4.7% (3.8%, 5.7%)		
Age (years)							NA		
5–18	0.7% (0.1%, 1.2%)	0.39 (0.15, 1.01)	0.30 (0.12, 0.80)	0.5% (0.0%, 1.2%)	0.53 (0.10, 2.92)	0.47 (0.08, 2.64)	0.7% (0.0%, 1.5%)	0.34 (0.11, 1.08)	0.27 (0.09, 0.86)
19–29	1.7% (0.8%, 2.5%)	1	1	1.0% (0.0%, 1.9%)	1	1	2.1% (0.9%, 3.3%)	1	1
30–39	4.3% (2.9%, 5.7%)	2.64 (1.43, 4.89)	2.40 (1.30, 4.46)	1.0% (0.0%, 2.0%)	1.02 (0.25, 4.12)	1.00 (0.25, 3.98)	6.4% (4.2%, 8.6%)	3.17 (1.58, 6.37)	2.87 (1.44, 5.72)
40–49	6.2% (4.4%, 8.0%)	3.91 (2.20, 6.96)	3.41 (1.90, 6.11)	4.1% (2.0%, 6.2%)	4.36 (1.41, 13.5)	4.22 (1.39, 12.8)	7.5% (4.9%, 10.1%)	3.78 (1.94, 7.40)	3.21 (1.65, 6.25)
50–59	5.8% (3.9%, 7.7%)	3.66 (2.12, 6.31)	3.01 (1.73, 5.23)	2.4% (0.8%, 4.1%)	2.55 (0.83, 7.89)	2.67 (0.85, 8.36)	8.2% (5.2%, 11.3%)	4.18 (2.23, 7.82)	3.12 (1.68, 5.79)
≥60	4.3% (2.7%, 5.8%)	2.65 (1.43, 4.90)	1.82 (0.98, 3.38)	1.2% (0.0%, 2.5%)	1.20 (0.27, 5.44)	1.11 (0.22, 5.51)	5.8% (3.6%, 8.0%)	2.86 (1.43, 5.70)	1.96 (0.99, 3.86)
Rural (vs urban)		3.01 (2.00, 4.55)	2.53 (1.62, 3.95)		NA	NA		NA	NA
Sex									
Female	3.2% (2.5%, 3.9%)	1	1	1.7% (0.9%, 2.5%)	1	1	4.1% (3.1%, 5.0%)	1	1
Male	4.0% (3.1%, 5.0%)	1.28 (0.95, 1.72)	1.52 (1.08, 2.14)	1.6% (0.8%, 2.3%)	0.94 (0.49, 1.77)	1.08 (0.56, 2.06)	5.5% (4.1%, 7.0%)	1.38 (0.98, 1.93)	1.67 (1.13, 2.48)
Household income									
0–20,000 Rupees	3.8% (3.1%, 4.5%)	1	1	1.8% (1.1%, 2.6%)	1	1	4.8% (3.8%, 5.7%)	1	1
>20,000 Rupees	2.5% (1.2%, 3.7%)	0.64 (0.38, 1.09)	0.95 (0.54, 1.66)	1.2% (0.4%, 2.0%)	0.65 (0.29, 1.44)	0.74 (0.32, 1.73)	4.7% (1.7%, 7.7%)	0.99 (0.50, 1.96)	1.06 (0.54, 2.10)
Education									
None/Primary	4.7% (3.6%, 5.8%)	1	1	1.7% (0.6%, 2.8%)	1	1	5.9% (4.4%, 7.3%)	1	1
Middle/Secondary	3.3% (2.6%, 4.1%)	0.70 (0.52, 0.95)	0.81 (0.58, 1.13)	2.0% (1.1%, 2.8%)	1.16 (0.53, 2.56)	1.19 (0.49, 2.85)	4.2% (3.1%, 5.3%)	0.74 (0.55, 0.99)	0.74 (0.51, 1.07)
Graduate	1.1% (0.3%, 1.8%)	0.21 (0.10, 0.46)	0.37 (0.16, 0.82)	0.8% (0.0%, 1.5%)	0.45 (0.13, 1.50)	0.54 (0.13, 2.19)	1.6% (0.0%, 3.3%)	0.31 (0.09, 1.04)	0.37 (0.13, 1.09)
Last injection given by									
Medical Doctor	2.1% (1.2%, 2.9%)	1	1	1.1% (0.3%, 2.0%)	1	1	3.1% (1.6%, 4.6%)	1	1
Registered Nurse/Medical Practitioner	4.4% (3.6%, 5.2%)	2.16 (1.37, 3.42)	1.56 (0.97, 2.53)	2.3% (1.3%, 3.4%)	2.08 (0.87, 4.99)	1.71 (0.71, 4.12)	5.3% (4.2%, 6.4%)	2.16 (1.28, 3.66)	1.56 (0.88, 2.75)
Other/Unknown	2.9% (1.7%, 4.0%)	1.38 (0.78, 2.45)	1.25 (0.70, 2.23)	0.9% (0.1%, 1.7%)	0.80 (0.25, 2.52)	0.71 (0.23, 2.21)	4.3% (2.5%, 6.2%)	1.60 (0.71, 3.58)	1.44 (0.73, 2.84)
Number injections received last 6 months	NA	1.02 (1.01, 1.03)	1.01 (0.99, 1.03)	NA	1.03 (0.99, 1.07)	1.02 (0.96, 1.08)	NA	1.01 (1.00, 1.03)	1.01 (0.98, 1.03)
Number of times donating blood	NA	0.99 (0.93, 1.05)	0.96 (0.87, 1.05)	NA	0.99 (0.91, 1.08)	0.97 (0.88, 1.07)	NA	1.01 (0.94, 1.08)	0.96 (0.85, 1.08)
Number of blood transfusions received	NA	1.36 (1.10, 1.69)	1.36 (1.10, 1.68)	NA	1.05 (0.69, 1.60)	0.99 (0.60, 1.64)	NA	1.56 (1.15, 2.10)	1.47 (1.10, 1.96)
Received a permanent tattoo									
No	3.4% (2.8%, 4.1%)	1	1	1.6% (1.0%, 2.2%)	1	1	4.5% (3.6%, 5.5%)	1	1
Yes	5.2% (3.1%, 7.3%)	1.54 (0.97, 2.45)	1.21 (0.74, 1.98)	2.2% (0.1%, 4.3%)	1.42 (0.49, 4.09)	1.36 (0.49, 3.82)	6.9% (3.9%, 10.0%)	1.57 (0.94, 2.63)	1.17 (0.67, 2.05)

**Table 3:** Weighted unadjusted and mutually adjusted odds ratios (OR) for having Hepatitis C antibodies (anti-HCV), by participant characteristics and risk factors overall and stratified by urban/rural status for adults age 18 or over.

	Total	Odds Ratio (95% Confidence Interval)		Urban	Odds Ratio (95% Confidence Interval)		Rural	Odds Ratio (95% Confidence Interval)	
Variables	Anti-HCV % (95% CI)	Unadjusted OR	Adjusted OR	Anti-HCV % (95% CI)	Unadjusted OR	Adjusted OR	Anti-HCV % (95% CI)	Unadjusted OR	Adjusted OR
Total	4.3% (3.6%, 5.1%)			1.9% (1.2%, 2.6%)			5.8% (4.7%, 6.9%)		
Age									
20–29	1.7% (0.8%, 2.5%)	1	1	1.0% (0.0%, 1.9%)	1	1	2.1% (0.9%, 3.3%)	1	1
30–39	4.3% (2.9%, 5.7%)	2.64 (1.43, 4.89)	2.43 (1.31, 4.51)	1.0% (0.0%, 2.0%)	1.02 (0.25, 4.12)	1.02 (0.26, 4.08)	6.4% (4.2%, 8.6%)	3.17 (1.58, 6.37)	2.89 (1.45, 5.77)
40–49	6.2% (4.4%, 8.0%)	3.91 (2.20, 6.96)	3.41 (1.90, 6.13)	4.1% (2.0%, 6.2%)	4.36 (1.41, 13.5)	4.36 (1.44, 13.2)	7.5% (4.9%, 10.1%)	3.78 (1.94, 7.40)	3.19 (1.64, 6.22)
50–59	5.8% (3.9%, 7.7%)	3.66 (2.12, 6.31)	2.96 (1.70, 5.16)	2.4% (0.8%, 4.1%)	2.55 (0.83, 7.89)	2.85 (0.92, 8.81)	8.2% (5.2%, 11.3%)	4.18 (2.23, 7.82)	3.03 (1.64, 5.63)
≥60	4.3% (2.7%, 5.8%)	2.65 (1.43, 4.90)	1.77 (0.95, 3.28)	1.2% (0.0%, 2.5%)	1.20 (0.27, 5.44)	1.13 (0.23, 5.68)	5.8% (3.6%, 8.0%)	2.86 (1.43, 5.70)	1.88 (0.95, 3.70)
Rural (vs urban)	NA	3.20 (2.09, 4.89)	2.59 (1.63, 4.11)	NA			NA		
Sex									
Female	3.6% (2.8%, 4.3%)	1	1	1.8% (0.9%, 2.7%)	1	1	4.6% (3.5%, 5.8%)	1	1
Male	5.2% (4.0%, 6.5%)	1.50 (1.10, 2.04)	1.73 (1.22, 2.44)	2.0% (1.0%, 3.0%)	1.12 (0.58, 2.15)	1.26 (0.66, 2.41)	7.3% (5.4%, 9.2%)	1.62 (1.15, 2.30)	1.89 (1.27, 2.81)
Household income									
0–20,000 Rupees	4.7% (3.8%, 5.5%)	1	1	2.2% (1.3%, 3.2%)	1	1	5.8% (4.7%, 7.0%)	1	1
>20,000 Rupees	2.8% (1.3%, 4.2%)	0.58 (0.34, 1.00)	0.96 (0.54, 1.70)	1.2% (0.3%, 2.0%)	0.52 (0.22, 1.21)	0.66 (0.27, 1.60)	5.7% (2.1%, 9.3%)	0.97 (0.49, 1.92)	1.11 (0.57, 2.21)
Education									
None/Primary	6.1% (4.6%, 7.5%)	1	1	2.2% (0.7%, 3.8%)	1	1	7.3% (5.5%, 9.1%)	1	1
Middle/Secondary	4.1% (3.1%, 5.0%)	0.66 (0.48, 0.90)	0.76 (0.54, 1.08)	2.3% (1.3%, 3.3%)	1.05 (0.45, 2.42)	1.21 (0.48, 3.06)	5.1% (3.8%, 6.5%)	0.69 (0.49, 0.97)	0.69 (0.47, 1.00)
Graduate	1.1% (0.3%, 1.8%)	0.17 (0.08, 0.36)	0.36 (0.16, 0.81)	0.8% (0.0%, 1.5%)	0.34 (0.10, 1.19)	0.59 (0.14, 2.48)	1.6% (0.0%, 3.4%)	0.21 (0.07, 0.63)	0.36 (0.12, 1.05)
Last injection given by									
Medical Doctor	2.3% (1.2%, 3.3%)	1	1	1.0% (0.1%, 1.9%)	1	1	3.6% (1.7%, 5.5%)	1	1
Registered Nurse/Medical Practitioner	5.3% (4.3%, 6.3%)	2.43 (1.48, 3.97)	1.75 (1.04, 2.92)	3.0% (1.6%, 4.1%)	2.95 (1.09, 7.98)	2.44 (0.89, 6.67)	6.4% (5.0%, 7.7%)	1.82 (1.02, 3.23)	1.61 (0.89, 2.90)
Other/ Unknown	3.6% (2.2%, 5.1%)	1.64 (0.90, 2.99)	1.45 (0.79, 2.66)	1.1% (0.1%, 2.1%)	1.13 (0.33, 3.95)	0.99 (0.28, 3.47)	5.7% (3.3%, 8.1%)	1.61 (0.81, 3.21)	1.56 (0.78, 3.14)
Number injections received last 6 months	NA	1.02 (1.00, 1.03)	1.01 (0.99, 1.03)	NA	1.02 (0.98, 1.07)	1.02 (0.97, 1.07)	NA	1.01 (0.99, 1.03)	1.01 (0.99, 1.03)
Number of times donating blood	NA	0.97 (0.89, 1.04)	0.95 (0.86, 1.04)	NA	0.98 (0.88, 1.08)	0.96 (0.87, 1.07)	NA	0.98 (0.90, 1.08)	0.95 (0.84, 1.07)
Number of blood transfusions	NA	1.32 (1.07, 1.63)	1.37 (1.11, 1.70)	NA	1.03 (0.67, 1.59)	1.01 (0.61, 1.65)	NA	1.48 (1.11, 1.97)	1.49 (1.10, 2.00)
Received a permanent tattoo									
No	4.2% (3.4%, 5.0%)	1	1	1.8% (1.1%, 2.6%)	1	1	5.6% (4.4%, 6.8%)	1	1
Yes	5.5% (3.3%, 7.7%)	1.35 (0.85, 2.14)	1.17 (0.71, 1.92)	2.4% (0.1%, 4.6%)	1.28 (0.44, 3.72)	1.32 (0.47, 3.71)	7.4% (4.2%, 10.7%)	1.35 (0.81, 2.27)	1.13 (0.65, 1.99)

**Table 4:** Weighted unadjusted and mutually adjusted odds ratios (OR) for having Hepatitis C antibodies (anti-HCV), by participant characteristics and risk factors overall for adults aged 40–59 years old.

	Total	Odds Ratio (95% Confidence Interval)	
Variables	Anti-HCV % (95% CI)	Unadjusted OR	Adjusted OR
Total	6.0% (4.6%, 7.4%)		
Setting			
Urban	3.3% (1.9%, 4.8%)	1	1
Rural	7.8% (5.7%, 9.9%)	2.48 (1.45, 4.25)	1.82 (1.01, 3.27)
Sex			
Female	5.4% (3.9%, 6.9%)	1	1
Male	6.8% (4.6%, 9.1%)	1.28 (0.84, 1.96)	1.63 (1.02, 2.61)
Household income			
0–20,000 Rupees	6.3% (4.8%, 7.9%)	1	1
>20,000 Rupees	4.8% (2.2%, 7.4%)	0.74 (0.40, 1.36)	1.23 (0.63, 2.39)
Education			
None/Primary	8.6% (6.2%, 11.0%)	1	1
Middle/Secondary	4.7% (3.0%, 6.5%)	0.53 (0.33, 0.83)	0.58 (0.36, 0.95)
Graduate	1.6% (0.0%, 3.3%)	0.17 (0.05, 0.55)	0.24 (0.06, 0.88)
Last injection given by			
Medical Doctor	2.9% (1.1%, 4.8%)	1	1
Registered Nurse/Medical Practitioner	7.9% (5.9%, 9.9%)	2.83 (1.43, 5.61)	2.14 (1.04, 4.40)
Other/Unknown	4.1% (1.8%, 6.3%)	1.40 (0.59, 3.32)	1.30 (0.54, 3.14)
Number injections received last 6 months	NA	1.01 (0.99, 1.03)	1.00 (0.98, 1.02)
Number of times donating blood	NA	0.91 (0.77, 1.08)	0.91 (0.75, 1.11)
Number of blood transfusions received	NA	1.35 (1.02, 1.79)	1.51 (1.18, 1.93)
Received a permanent tattoo			
No	5.9% (4.5%, 7.3%)	1	1
Yes	7.6% (2.4%, 12.8%)	1.30 (0.60, 2.82)	1.03 (0.43, 2.47)

## Discussion

This study, the first assessing the prevalence and risk factors for HCV infection in Punjab, India, found an overall weighted prevalence of anti-HCV of 3.6% and HCV RNA of 2.6%. We found that males, persons aged 40–59, and persons living in rural areas had the greatest odds of being infected with HCV. Additionally, HCV infection was more common among those who lacked education, received a blood transfusion, and had their last injection given by a nurse or other medical practitioner as compared to a medical doctor. Through multivariable analysis, we found no increased likelihood of being anti-HCV positive with increases in the reported number of participants’ medical injections.

The association of HCV with age and rural residence has been observed in previous studies from Punjab.[24] Also consistent with our analysis, studies from other countries have identified a particular age or birth cohort with a high prevalence of HCV compared to others.[25, 26] This cohort effect is demonstrated by persons born between 1945 and 1965, so called “Baby Boomers” in the United States.[27, 28] In the United States, the higher HCV prevalence among Baby Boomers has been attributed largely to injection drug use during their youth, the lack of an HCV screening test for blood and blood products prior to 1990, and to the effect of the HIV epidemic, recognized during the 1980s.[27, 28] In our study, noting the increasing prevalence with age, it would be tempting to consider that transmission risk has decreased over time and younger people are at lower risk, however, the youngest age groups studied, 5–18 and 19–29 year olds, had HCV seropositive rates of over 1% and 2% respectively, suggesting that transmission risk persists in Punjab. In fact, a rise in prevalence of injection drug use in Punjab has been described among teens and the youth population[29] and may present an emerging risk for HCV infection in similarly aged populations in the years to come. Very few admitted to injection drug use in our survey, which may reflect social desirability bias on the part of participants.

Residence in a rural versus urban area was determined to be an effect modifier in our analysis. Individuals in rural areas of Punjab had 2.5 times the odds of being anti-HCV positive as those in urban settings after adjusting for covariates, a result comparable to other studies in North India[30]. Upon stratification, we found that sex, age and blood transfusions were associated with HCV among participants in rural areas, whereas in urban areas the only significant association was with age. Poverty was not associated with infection. There is a paucity of trained healthcare professionals in rural areas of Punjab, so healthcare in those regions is often delivered by unqualified practitioners who may adopt unsafe injection practices,[31] possibly contributing to the elevated prevalence of HCV among rural residents in Punjab in our study.

The finding that blood transfusions were a risk factor for HCV highlights the need for improved blood safety practices in Punjab. Mandatory testing for HCV was implemented in blood banks in India in 2002.[32] However, participants in our study who received their first transfusion in 2002 or later were no less likely to be anti-HCV positive than those who received transfusions before mandatory testing began. Despite the existence of statewide blood safety guidelines, an association between receipt of a blood transfusion and having HCV infection persisted in our study, regardless of when the blood transfusions were received, and may suggest a persistent mode of HCV transmission in Punjab. These findings underscore the need for greater enforcement and monitoring of blood banks to ensure proper testing procedures are followed to prevent transmission in these settings.

Previous studies have also found inadequate infection control practices among healthcare workers in India,[33, 34] however, the number of medical injections was not associated with HCV after adjusting for covariates in our study. It is important to note that in a cross-sectional study, to identify associations with medical practices is challenging. However, we found an increased likelihood of being anti-HCV positive among those who received their last injection from someone other than a medical doctor, a finding which was not specific to rural areas. A 2002 study in Punjab found that a considerable percentage of physicians with knowledge of parenteral HCV transmission risk nevertheless reused needles and syringes with their patients.[35] Furthermore, throughout India and in Punjab, treatment with injectable medicine is perceived to be the treatment that ensures rapid therapeutic relief.[36] This belief has been inculcated over many years by physicians themselves, and there are financial incentives to deliver treatment through a “procedure”, such as an injection.[36] Although increased availability of disposable syringes helps temper these risks, healthcare workers throughout Punjab could benefit from further training on safe injection practices to prevent the spread of HCV and other diseases.

The prevalence of chronic HCV infection found in our study was slightly less than was determined by a 2012 study in the region,[19] but in a population of roughly 28 million, still translates to nearly three quarters of a million people chronically infected in the state of Punjab alone. Future screening efforts need to address this burden of disease to identify infected individuals and link them to care and treatment. The results of this study can be used to target screening and linkage to care efforts in the state, to ensure the highest yield of HCV infected individuals. Screening efforts in Punjab should target rural districts and persons age 30 and older. India’s expenditure on health care as a percentage of its gross domestic product (1.3% in 2015–2016) is among the lowest in the world, and the country has no system to monitor patients.[37] Nationwide surveillance of hepatitis is also lacking in the country and focuses primarily on hepatitis A and E.[38] Testing for incident HCV and HBV cases is only supported by the country’s national Integrated Disease Surveillance Programme (IDSP) in outbreak situations.[38] Fortunately, treatment costs for HCV infection in India have decreased significantly with the introduction of direct acting antiviral drugs in 2015, which have proven to be highly effective.[39] In 2016 Punjab became the first state in India to make the commitment to treat HCV patients free of charge.[40, 41] Through July 2017, over 32,000 patients have been treated through the program,[40] representing an important step in control of the disease. However, access to treatment alone cannot end the epidemic of HCV globally or in Punjab. More initiative is needed with respect to disease awareness, diagnosis, and prevention. Nevertheless, with treatment options becoming more effective, affordable, and available to patients, there is hope that Punjab could be reaching a turning point to mitigate the burden of HCV.

Our study is subject to several limitations. First, we were not able to independently verify any of the responses on the questionnaire. Also, we could not determine the number of non-responders, though it was reported from the field that interest was very high and 98% of households participated. As with any cross-sectional study that examines a chronic condition, it is challenging to attribute risk due to lack of temporality, as the HCV infection could have occurred at any time during the lifetime of the study subjects. The sampling method of this study, which included multiple participants from a single household, could lead to potential selection bias. Persons living together are more likely to exhibit similar behaviors and could lead to disproportionate risks in the sample that may not be representative of the greater population. Additionally, the sampling method of our study was not designed to produce precise per-region prevalence estimates; there was a preponderance of people surveyed from rural areas in districts that were found to have a high prevalence of HCV. These results should be interpreted with caution, as this could lead to overestimation of the prevalence in these areas. The face-to-face nature of the questionnaire creates the potential for social desirability bias. Injection drug use is a significant risk factor for HCV, but self-report of this behavior was extremely low (0.09%) among participants in our study despite reports of worrisome trends of increased injection drug use in the state.[22] The number of persons associated with some of the exposures and risk factors, notably dialysis (n = 26) and injection drugs (n = 5), was small, making associations of these risk factors with HCV seropositivity difficult to determine. Thus an important risk behavior may be substantially underrepresented in this analysis. Finally, we cannot rule out false positive anti-HCV among those that tested negative for RNA.

Population studies provide critical data for planning control efforts of HCV. The effectiveness and decreasing costs of DAAs are bringing control efforts within reach in many countries.[9] Programs that target screening and linkage to care for the highest risk populations in addition to addressing ongoing transmission risk, are likely to be the most successful and cost effective. Population serosurveys, such as the study in Punjab presented here, can address key information gaps and inform policy makers in efforts to alleviate the public health burden of HCV infection across afflicted regions worldwide.[42]
